# Remodeling of central metabolism in invasive breast cancer compared to normal breast tissue – a GC-TOFMS based metabolomics study

**DOI:** 10.1186/1471-2164-13-334

**Published:** 2012-07-23

**Authors:** Jan Budczies, Carsten Denkert, Berit M Müller, Scarlet F Brockmöller, Frederick Klauschen, Balazs Györffy, Manfred Dietel, Christiane Richter-Ehrenstein, Ulrike Marten, Reza M Salek, Julian L Griffin, Mika Hilvo, Matej Orešič, Gert Wohlgemuth, Oliver Fiehn

**Affiliations:** 1 Institute of Pathology, Charité University Hospital, 10117 Berlin, Germany; 2 Research Laboratory of Pediatrics and Nephrology, Hungarian Academy of Sciences, Budapest, Hungary; 3 Interdisciplinary Breast Center, Charité University Hospital, 10117 Berlin, Germany; 4 Institute of Pathology, DRK Kliniken Berlin, 12559 Berlin, Germany; 5 Department of Biochemistry, University of Cambridge, Cambridge, CB2 1GA, United Kingdom; 6 VTT Technical Research Centre of Finland, Espoo, Finland; 7 Genome Center, University of California Davis, Davis, CA, USA

**Keywords:** Breast cancer, Metabolomics, Gas chromatography, Mass spectrometry, Cancer detection

## Abstract

**Background:**

Changes in energy metabolism of the cells are common to many kinds of tumors and are considered a hallmark of cancer. Gas chromatography followed by time-of-flight mass spectrometry (GC-TOFMS) is a well-suited technique to investigate the small molecules in the central metabolic pathways. However, the metabolic changes between invasive carcinoma and normal breast tissues were not investigated in a large cohort of breast cancer samples so far.

**Results:**

A cohort of 271 breast cancer and 98 normal tissue samples was investigated using GC-TOFMS-based metabolomics. A total number of 468 metabolite peaks could be detected; out of these 368 (79%) were significantly changed between cancer and normal tissues (p<0.05 in training and validation set). Furthermore, 13 tumor and 7 normal tissue markers were identified that separated cancer from normal tissues with a sensitivity and a specificity of >80%. Two-metabolite classifiers, constructed as ratios of the tumor and normal tissues markers, separated cancer from normal tissues with high sensitivity and specificity. Specifically, the cytidine-5-monophosphate / pentadecanoic acid metabolic ratio was the most significant discriminator between cancer and normal tissues and allowed detection of cancer with a sensitivity of 94.8% and a specificity of 93.9%.

**Conclusions:**

For the first time, a comprehensive metabolic map of breast cancer was constructed by GC-TOF analysis of a large cohort of breast cancer and normal tissues. Furthermore, our results demonstrate that spectrometry-based approaches have the potential to contribute to the analysis of biopsies or clinical tissue samples complementary to histopathology.

## Background

In a recent update, Hanahan and Weinberg added reprogramming of energy metabolism to the list of hallmarks of cancer [1]. Historically, cancer was regarded as metabolic disease long before being decoded as disease of genes and mutations. More than 80 years ago, Otto Warburg reported an increased anaerobic glycolysis in cancer cells compared to normal cells [2]. Additional to the Warburg effect [3,4], cancer cells exhibit increased protein and nucleotide synthesis [5,6], increased fatty acid synthesis and changes in fatty acid metabolism [7-9]. Integrating data of different sources, models of the altered metabolism in cancer were developed, see for example [4,10,11]. In recent years, there has been a renewed interest in the altered metabolism of cancer cells coupled with progress in development of new metabolic drugs [12]. In order to obtain a comprehensive view on the metabolic changes between invasive carcinomas and normal breast tissues, herein a metabolomics study of breast cancer was carried out with a focus on the low molecular weight molecules of central metabolism. In a separate project, a breast cancer cohort was investigated by lipidomics with a focus on altered membrane lipid metabolism [13].

Analytical chemistry methods allow the investigation of the metabolic changes that occur in cancer tissues. Using gas chromatography (GC), liquid chromatography (LC) or capillary electrophoresis (CE) coupled with mass spectrometry (MS) hundreds of molecules in a tissue sample can be analyzed simultaneously. In two preceding studies using gas chromatography followed by time-of-flight mass spectrometry (GC-TOFMS) we successfully profiled ovarian and colon cancer [14,15]. This approach, allowed the monitoring of hundreds of small molecules with masses of up to 500 Da. Using spectral libraries like BinBase [16] many of these metabolite peaks can be mapped to metabolites with known chemical structures and functions.

Breast cancer is a public health issue of global relevance with more than one million new cases diagnosed annually and more than 400,000 death cases worldwide [17]. After surgery, adjuvant chemotherapy is offered to most breast cancer patients to reduce the risk of relapse. However, about 40% of the early breast cancer patients have a low risk of developing distant metastases and of dying because of the disease [18]. On the other hand, aggressive subtypes like triple-negative breast cancer have a poor prognosis and are difficult to treat [19]. New targeted therapies including small molecule inhibitors and therapeutic antibodies are currently under development and being tested in clinical trials [20], however many of the new approaches achieved only limited response rates. Therefore, as a step to personalized medicine, a better understanding of the functional pathway alterations in breast cancer is needed to avoid over-treatment and select patients for individualized and targeted therapies.

GC-TOFMS based metabolomics provides a wide coverage of the central part of the cellular metabolism including glycolysis, citrate cycle, amino acid and nucleotide metabolism. These pathways are altered in cancer cells and can be targeted by metabolic drugs. Herein, we report on the comparison of 275 invasive breast cancer samples with 94 normal tissue samples using GC-TOFMS. The purpose of this study is two-fold: (i) to analyze the metabolic changes in the central pathways between invasive carcinoma and normal breast tissues on a global scale and (ii) to identify key metabolic markers that separate cancer from normal tissues with high sensitivity and specificity.

## Results

### Metabolomics analysis of breast cancer

The entire cohort of breast cancer and normal tissues was divided into a training set (TS, 226 samples, of which 184 were tumor samples) and a validation set (VS, 143 samples, of which 87 were tumor samples). Analysis of the GC-TOFMS spectra of the TS samples led to the detection of 468 most abundant metabolite peaks that were present in breast cancer tissues. Subsequently, these metabolites were measured in the VS. 162 of the detected metabolite peaks could confidently be mapped to known chemical structures and metabolite names.

**Figure 1:**
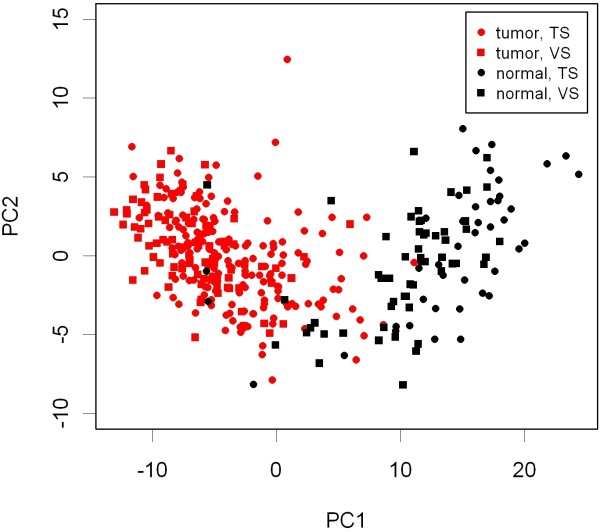
** Principal components analysis (PCA) of the GC-TOFMS data.** The rotation matrix that connects the 162 identified metabolites with the principal components (PCs) was determined using the TS data. In the TS, the 1^st^ PC captured 34.9% of the total variance; in the VS the 1^st^ PC captured 34.6% of the total variance. The 1^st^ PC was significantly decreased in cancer compared to normal tissues in TS (p = 2.2E-22) and VS (p = 8.4E-41).

Using unsupervised analysis methods we investigated the contribution of the malignancy of the tissues to the total variance of the dataset. The results of a principal components analysis (PCA) of TS and VS are shown in Figure 1. In the TS, the first two PCs captured 34.9% and 5.2% of the total variance of the metabolomics data. In the TS, the 1^st^ PC was significantly decreased in cancer compared to normal tissues (p = 2.2E-22). In the VS, the first two PCs captured 34.6% and 3.5% of the total variance. Further, the 1^st^ PC could be validated as being decreased in cancer compared to normal tissues (p = 8.4E-41). In summary, in TS and VS, the 1^st^ PC captured more than one-third of the total variance of the metabolomics data and correlated strongly with the malignancy of the studied tissue samples.

### Metabolic changes between breast cancer and normal tissue

Metabolite-by-metabolite analysis of the training set (TS) led to detection of 427 significantly changed metabolites between cancer (T) and normal tissues (N). Out of these, 363 (85%) remained significant after Bonferroni correction and 368 (86%) could also be validated by analysis of the validation set (VS). Among the validated metabolites, 247 were decreased and 121 increased in tumors, corresponding to 53% and 26% of the entire set of metabolites detected by GC-TOFMS.

**Figure 2:**
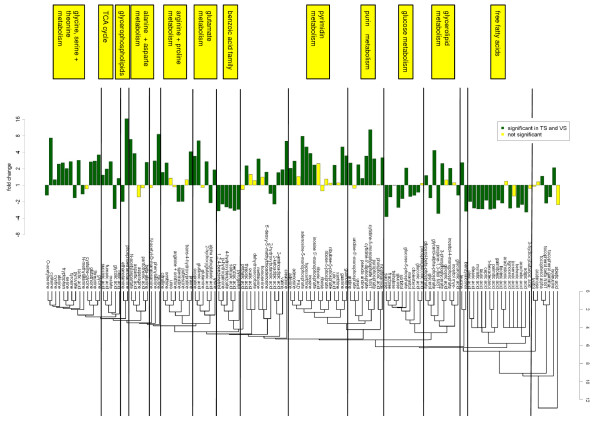
** PROFILE clustering of 129 KEGG metabolites with fold changes between cancer and normal tissues.** Ordering of the metabolites along the x-axis reflects the distance of metabolites in the network of enzymatic reactions. Significant metabolite changes in the TS that could be validated in the VS (green) and not significant or not validated metabolite changes (yellow).

For functional analysis, PROFILE clustering [14] was used to order the metabolites according to their mutual proximity in the metabolic network. Figure 2 shows the fold changes of 129 metabolites that are present in the KEGG data base. By proceeding through the cluster map form left to right, the metabolic changes include up-regulation of many amino acids, changes in TCA cycle, changes in glycerophospholipid metabolism, down-regulation of the benzoic acid family, up-regulation of most of the nucleotides and their phosphates, down-regulation of the sugar cluster including sucrose, fructose and glucose, and down-regulation of most of the free fatty acids.

**Figure 3:**
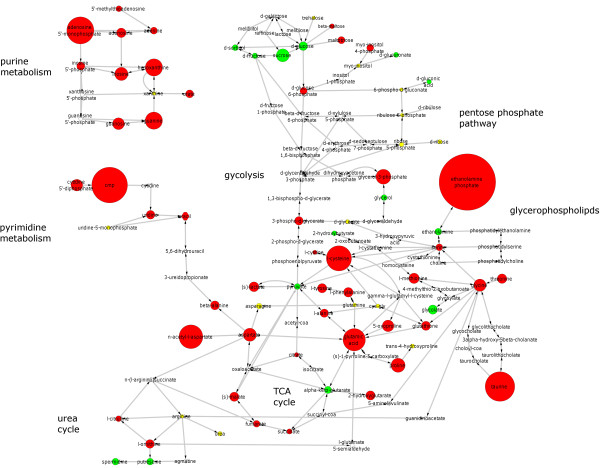
** Metabolic map of breast cancer showing alterations in energy metabolism, catabolism of amino acids, nucleotide metabolism and other pathways.** Networks were generated using Cytoscape with the Metscape plug-in. Significantly increased metabolites in tumor compared to normal tissue (red nodes), significantly decreased metabolites (green nodes), unchanged metabolites (yellow nodes) and metabolites not in the GC-TOFMS screen (white nodes). The size of the nodes is proportional to the absolute value of the fold change.

A metabolic network was constructed using Cytoscape with MetScape plug-in (Figure 3). Two metabolites were connected by an edge, if they can be converted into each other by an enzymatic reaction. In breast cancer tissues, glucose and other sugars were decreased while intermediates of the glycolysis pathway such as glucose-6-phosphate and 3-phospho-glycerate were increased. The equilibrium between pyruvate and lactate was shifted towards lactate. Many compounds of the TCA cycle were increased, with the exception of alpha-keto-glutarate that was decreased in cancer tissues.

Strong deregulation occurred in glutamate metabolism with a shift of equilibrium from alpha-ketoglutarate (fold change = -2.1, p = 2.4E-17) towards glutamate (fold change = 6.5, p = 1.6e-40). Nineteen proteinogenic amino acids (all except histidine) could be detected by the GC-TOFMS approach. Out of these 16 were strongly increased in the cancer tissues (all fold changes >1.9, all p-values <5.0e-10). Only one of the detected proteinogenic amino acids, asparagine, was decreased between cancer a normal tissues (fold change = -1.6, p = 1.5e-07), while glutamine and arginine remained unchanged.

Amphiphilic phospholipids are the building blocks of the cell membrane and are synthesized from choline and ethanolamine via the Kennedy pathway. Within this pathway, we have detected a shift of the equilibrium from ethanolamine (fold change = -2.0, p = 0.00041) to phosphoethanolamine (fold change = 16.3, p = 1.8e-44). Nucleotides, nucleosides and their phosphates were generally increased in the cancer tissues with the strongest regulations belonging to CMP (fold change = 10.3, p = 1.4e-57) and AMP (fold change = 7.8, p = 3.4e-49).

### Metabolite based separation of cancer and normal tissues

**Figure 4:**
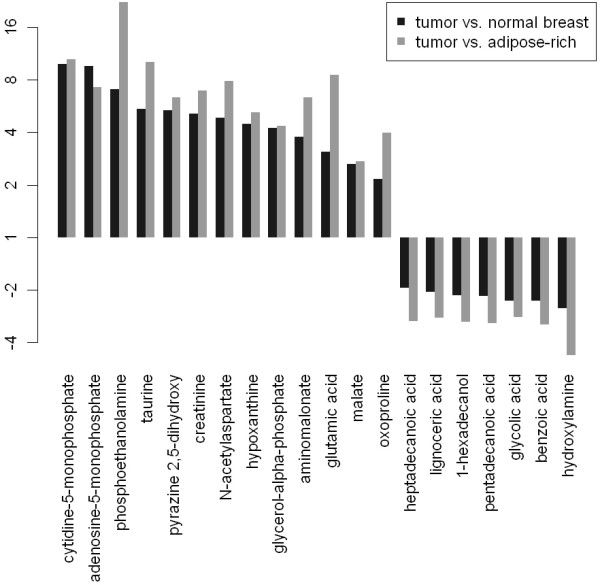
** Fold changes of 13 tumor markers and 7 normal tissue markers.** Each marker separates tumor (T) from normal breast (NB) and from adipose-rich normal tissues (NA) with sensitivity and specificity >80%.

In order to develop a metabolite-based molecular approach for the detection of breast cancer, we analyzed each metabolite for its classifying power. 50 metabolites, of which 20 have a known chemical structure, separated tumor (T) from normal breast tissues (NB) as well as tumor from normal adipose-rich tissues (NA) with sensitivity and specificity >80%. The fold changes of these marker metabolites are shown in Figure 4. We found 13 tumor markers (increased in T) and 7 normal tissue markers (increased in NB and NA).

**Figure 5:**
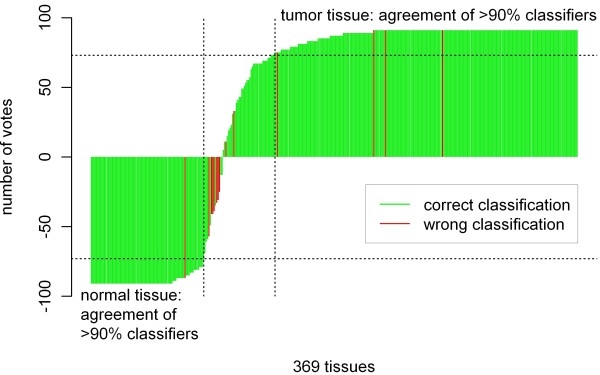
** Metabolite based prediction of the malignancy of 369 tissues.** 91 classifiers are constructed from the 20 most discriminatory metabolites that are shown in Figure 4. The bars show the results of an equal voting of the 91 classifiers. A majority decision predicts the malignancy with high accuracy (sensitivity = 97.0%, specificity = 93.9%).

In order to enhance the separation, each of the tumors markers was divided by each of the normal tissues markers yielding 13 × 7 = 91 metabolite ratios that served as classifiers. For each classifier, we defined a cut-off value for the classification in cancer and normal tissues by maximizing the sum of sensitivity and specificity. Then, we applied a voting system counting each of the classifiers above the cut-off +1, and each of the classifier below the cut-off -1. The votes for 91 classifiers were summed and the results are shown in Figure 5. A strong correlation was observed between the votes for different classifiers: For 217 tissue samples (58.8%) there was unisonous agreement among all classifiers, for 319 tissue samples (86.4%) more than 90% of the classifiers agreed on malignancy status.

Next, we compared the classification result obtained by the majority of the classifiers with the histopathological classification that is the gold standard for cancer detection. Only 8 out of 271 tumors and 6 out of 98 normal tissues were classified incorrectly, leading to a sensitivity of 97.0% and a specificity of 93.9% of the molecular test. Further, restricting the analysis to the 319 tissues where more than 90% of the classifiers agreed, only one tumor and four normal tissues were classified incorrectly, leading to a sensitivity of 99.6% and a specificity of 95.5%.

**Figure 6:**
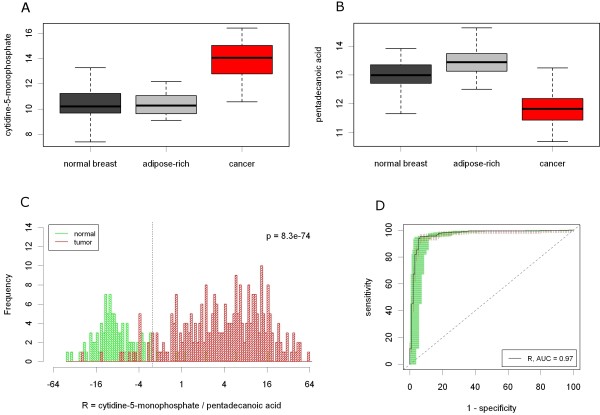
** Prediction of tissue malignancy by the ratio R = cytidine-5-monophosphate / pentadecanoic acid.** (**A**) Up-regulation of cytidine-5-monophosphate in cancer compared to normal tissues. (**B**) Down-regulation of pentadecanoic acid in cancer compared to normal tissues. (**C**) Histogram showing the distribution of the ratio R in cancer and normal tissues (x-axis: log2 scale). R was significantly higher in cancer than in normal tissues (p = 8.3e-74, Welch’s t-test). A cut-off at R = 0.39 maximizes the sum of sensitivity and specificity. (**D**) ROC curve of R with confidence intervals for sensitivity (red lines) and specificity (green lines). The cut-off leads to at sensitivity of 94.8% and a specificity of 93.9% for the separation of cancer from normal tissues.

Among all classifiers the ratio of cytidine-5-monophosphate / pentadecanoic acid had the highest significance for changing between cancer and normal tissues (T vs. N: p = 8.3E-74) and at the same time had the highest significance for changing between malignant and normal breast tissues (T vs. NB: p = 7.8E-14). Maximizing the sum of sensitivity and specificity lead to a cut-off value = 0.39. Using this cut-off point, only 14 out of 271 tumors and 6 out of 98 normal tissues were incorrectly classified leading to 94.8% sensitivity and 93.9% specificity. Histogram and ROC curve of the classifier are shown in Figure 6.

### Nearest centroid classification

We investigated the performance of classifiers including 2, 3, …, 162 metabolites with known structure for the separation of cancer from normal tissues. To estimate sensitivity and specificity of the classifiers, we combined nearest centroid classification with a multiple random validation strategy, as in [21]. It turned out that on average a sensitivity of about 96% and a specificity of about 94% was obtained independent of the number of metabolites. The classification results did not improve when a larger number of metabolites was used for classification (Table 1 and data not shown).

**Table 1:** Prediction of tissue malignancy by metabolite ratios and by nearest centroid classification

**method**	**# of metabolites**	**sensitivity**	**specificity**
metabolite ratios: CMP / pentadecanoic acid	2	94.8%	93.9%
metabolite ratios: majority vote of 91 classifiers	20	97.0%	93.9%
cancerclass: nearest centroid	2	95.1% (86.4% - 97.6%)	93.9% (81.8% - 100%)
cancerclass: nearest centroid	20	97.1% (94.2% - 99.1%)	93.9% (84.8% - 100%)
cancerclass: nearest centroid	162	95.1% (93.2% - 97.1%)	93.9% (84.8% - 100%)

CMP : cytidine-5-monophosphate.The metabolite ratio method was developed in the current work. Nearest centroid classification was executed using the R package cancerclass [37]. For the latter method, classification rates including 95% confidence intervals were estimated using multiple random validation.

## Discussion

Our results show that tissues collected during breast surgery have reproducible metabolite profiles that can be analyzed by using GC-TOFMS. Using this approach, 478 metabolite peaks could be detected and quantified; 79% of these were changed between cancer and normal tissues. Thus, there is a difference in the concentrations of many metabolites between cancer and normal tissues including changes as high as tenfold and more for some metabolites. The high rate of validated metabolites in a predefined training-validation analysis, even after the conservative Bonferroni correction, shows that the GC-TOFMS is a robust approach to detect the metabolite changes associated with malignant progression. Data acquisition at different time points is considered as a main source of variance in many GC-MS studies. However, the high degree of reproducibility (86% of metabolic changes) between training and test data set underscores the robustness GC-TOFMS platform and of the detected metabolic changes in the current study.

A metabolic map of breast cancer was constructed by visualizing the metabolite changes in the metabolic network including pathways like glycolysis, TCA cycle, nucleotide metabolism and catabolism of amino acids. The metabolic map can serve as a tool for hypothesis building about the metabolic processes in breast cancer and help to develop strategies for the therapeutic targeting of metabolism in cancer cells. Several metabolic drugs, some of them targeting the central energy metabolism, are currently under development and investigated in clinical studies [12].

Cancer is commonly considered as a genetic disease that is driven by mutations of oncogenes and tumor suppressor genes. However, one of the major underlying purposes of those genetic and gene expression changes is to create a metabolic phenotype for cancer cells that is essential for tumor cell growth and survival [8,22]. The metabolic phenotype of cancer includes alterations in glycolysis, amino acid metabolism, nucleotide metabolism and glycerophospholipid metabolism that were confirmed in the current study.

Glutamine, the most abundant amino acid in blood plasma, can be metabolized to pyruvate and lactate through glutamate, alpha-keto-glutarate and via the citric acid cycle. This process, termed glutaminolysis is an important source of energy, carbon and nitrogen in cancer cells [23]. In the present study, we have observed strong regulation of this pathway including a strong up-regulation of glutamate (fold change = 6.5) while glutamine was unchanged. This suggests that glutamine metabolism and in particular glutaminase, the enzyme that converts glutamine to glutamate, should be a potential target for intervention. In the 1980s three glutamine analogs raised great expectations as possible antineoplastic agents. But the promising results obtained in model systems could not be confirmed in clinical trials due to dose limiting side effects and ineffectiveness of treatment [24]. However, recently a small molecule inhibitor was shown to target GLS (kidney-type glutaminase) and to have antiproliferative activity in breast cancer cells while being unharmful to normal cells [25]. Furthermore, a connection between MYC (v-myc myelocytomatosis viral oncogene homolog), a master transcription factor and oncogene that is deregulated in many cancers and glutamine metabolism has been described [11]. MYC is known to be amplified in about 5% of breast cancers and associated with a more aggressive subtype and shortened survival [26].

In the GC-TOFMS metabolomics based approach we observed a two- to four-fold down-regulation of almost all detected free fatty acids (Figure 2). The down-regulation of fatty acids seems to contradict the up-regulation of fatty acid synthase (FASN) and increased *de novo* fatty acid synthesis that is found in many cancers [5,9]. Interestingly, a lipidomics study of breast cancer showed an up-regulation of many membrane lipids in cancer compared to normal tissues [13]. Thus, the metabolomics described in this paper together with the previously reported lipidomics data support the hypothesis that *de novo* fatty acid synthesis is potentially increased in breast cancer, but free fatty acids are rapidly metabolized to synthesize membrane phospholipids.

**Figure 7:**
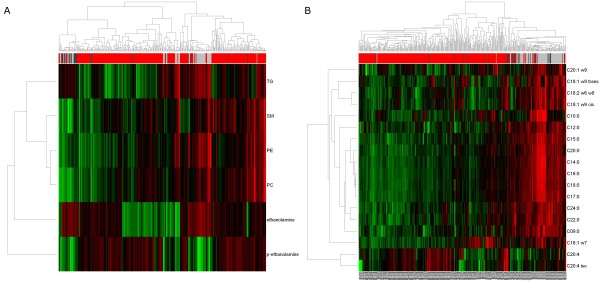
**Heatmaps of glycerophospholipids and free fatty acids.** (**A**) Heatmap of ethanolamine and phospho-ethanolamine, both detected by GC-TOFMS, and the total amounts of phosphatidylethanolamine (PE), phosphatidylcholines (PC), spingomylins (SM) and triglycerides (TG), all detected by UPLC-MS. (**B**) Heatmap of 18 free fatty acids, all detected by GC-TOFMS. The fatty acids are annotated by the number of carbon atoms, the number of double bonds and the omega position of the double bonds. The bars at the top of the heatmaps encode the type of tissue: invasive carcinoma (T) = red, normal breast tissue (NB) = dark grey, adipose-rich normal tissue (NA) = light grey.

The GC-TOFMS data also showed a shift of the equilibrium from ethanolamine that was decreased to phospho-ethanolamine that was highly increased in the cancer tissues possibly indicating a stimulation of the Kennedy pathway. To correlate these changes with the content of membrane lipids, we have extracted the total content of different kinds of membrane lipids from the UPLC-MS data published before [13]. However, there were neither pronounced correlations between phospho-ethanolamine and the total content of phosphatidylethanolamine (PE) nor between phospho-ethanolamine and the total content of phosphatidylcholine (PC), see Figure 7A. The mechanism behind might be that, in tumors tissues, the Kennedy pathway is regulated in such a way, that a sufficient concentration of phospho-ethanolamine is always available. In fact, the same choline kinases that catalyse the reaction of choline to phospho-choline also catalyse the reaction of ethanolamine to phospho-ethanolamine. Choline kinases were detected to be up-regulated in tumors and represent potential targets for therapeutic intervention [27].

In order to develop a classifier that separates between cancer and normal tissues, we identified 13 increased metabolites and 7 decreased metabolites that separated cancer from normal tissues with sensitivity and specificity >80%. We did not detect any perfect biomarker in the sense that a single metabolite was abundant and specific to cancer tissue, but absent in normal tissues or *vice versa*. Therefore, we built ratios of metabolites in order to construct scale-invariant tissue classifiers that are independent of the amount of tissue under investigation and do not depend on data normalization. These two-metabolite classifiers separated cancer from normal tissues with high sensitivity and specificity and had a high agreement among each other. In an extensive additional analysis we investigated the performance of classifiers that include a larger number of metabolites using a multiple random validation strategy. It turned out that classifiers including more metabolites did not outperform classifiers including only two metabolites (Table 1). This result underscores the relevance of the two-metabolite ratio classifiers as a promising strategy for diagnostic classification.

The metabolite ratio cytidine-5-monophosphate/pentadecanoic acid showed the strongest discrimination between cancer and normal tissues and permitted detection of cancer with a sensitivity of 94.8% and a specificity of 93.9%. Pentadecanoic acid (C15:0) is a known fatty acid present in milk and it has been found to be a good marker of milk fat intake when found in serum. However, a heatmap of the free fatty acids shows a high correlation of the free fatty acids C14:0, C15:0, C16:0, C17:0, C18:0, C20:0 in the METAcancer cohort (see Figure 7B, all Pearson correlations >0.82). Thus, in the classifier, it is possible to replace pentadecanoic acid by some other saturated fatty acids. Indeed, classifiers including ligoneric acid (24:0) or heptadecanoic acid (17:0) instead of pentadecanoic acid to a large extend coincide with the classifiers that include pentadecanoic acid.

For the detection of metabolite changes, we worked with a predefined training set (TS) and validation set (VS). All of the 20 most discriminatory metabolites are among the validated changed metabolites between cancer (T) and normal tissues (N). It should be noted, though, that normal breast tissue might consist of normal epithelium as well as various amounts of adipose tissue. Therefore, it was important to distinguish different kinds of normal tissues (NA and NB) and to develop a classifier that is independent of the fat content of the tissue. Thus, only metabolites that separated cancer from both, normal breast (NB) and normal adipose-rich tissues (NA) were included in the final classifier. This part of the analysis could only be performed in the pooled TS and VS due to a low number of normal breast samples. Another limitation of the current study is its retrospective character and the use of preselected samples that were enriched for cancer or normal cells. The cancer classifiers developed here need to be further validated using random samples from biopsying or surgery. In doing so, concordance with histopathology (accuracy) and reproducibility in repeated analyses of the same tissue (precision) of the classifiers should be further evaluated.

A critical factor determining whether a method is applicable for routine cancer diagnostics, for example during surgery, is the time needed for sample analysis. In order to cope with the inherent heterogeneity nature of the tissues, it is necessary to probe various areas of the tissue and still keep the total analysis time in the range of minutes. There is recent progress in automation and miniaturization of MS devices and such tools are expected to become cheaper and easier to handle during the next few years [28,29]. First hand-portable, automated GC-MS devices is commercially available today [30]. Additionally, an automated LC/CE-MS device in the size of a personal computer is being developed in the European ARROWS FP7 project [31]. Automation of sample acquisition, preparation and data generation will be important to translate MS based tissue investigations approaches into clinical applications.

## Conclusions

For the first time, a metabolic map of breast cancer was constructed by GC-TOF analysis of a large cohort of breast cancer and normal tissues. In future, MS based devices could contribute to the analysis of biopsies and surgical samples. While metabolite analyses will not replace microscopic evaluation, metabolomics could be used complementary to histopathology, e.g. for an initial quality control of biopsies directly after sampling or as additional assessment method for biopsies or surgical samples.

## Methods

### Study cohort

The study cohort consisted of 369 fresh frozen breast tissues that were collected at the Charité Hospital in the tissue bank of the European FP7 METAcancer consortium. The project was approved by the institutional review board of the Charité Hospital (EA1/139/05). For histopathological quality control, an H&E section of each frozen tissue was evaluated by a pathologist. 271 samples contained cancer tissue and had a tumor area ≥40% (T), the remaining 98 samples contained exclusively normal tissue (N). The normal tissues included 27 samples with ≥40% normal breast tissue (NB) and 71 adipose-rich samples with <40% normal breast tissue (NA).

As a basis for a predefined training-validation strategy, the entire cohort was divided in a training set (TS) of 184 + 42 (T + N) samples and a validation set (VS) of 87 + 56 samples. In detail, tumor samples were split randomly in a way that training (2/3) and validation (1/3) sets did not differ with respect to the following parameters: tumor stage, tumor grade, age (≤50 and >50 years) and estrogen receptor status. To this end, 100,000 splits were randomly drawn and the one with the lowest Kullback-Leibler divergence was chosen. All 42 normal tissues in the TS were paired with tumor tissues in the TS from the same patients. The VS included 24 normal tissues that were paired with tumor tissues and 32 additional unpaired normal tissues.

### GC-TOFMS based metabolomics

GC-TOFMS data acquisition of the 369 tissues was performed as previously published [16], following the guidelines of the metabolomics standard initiative [32]. Briefly, 20 mg frozen breast tissue samples were homogenized and extracted with 1 ml degassed isopropanol/acetonitrile/water (3/3/2) at 4°C for 5 min. The extracts were subsequently dried down and re-suspended in 50% aqueous acetonitrile to remove most of the complex lipids. After dry evaporation, extracts were derivatized and subjected to GC-TOFMS (Leco Pegasus IV) fitted with automatic liner exchange-cold injection (Gerstel). Raw data were deconvoluted using ChromaTOF (Leco) and mass spectra were exported for further data processing by the BinBase database, including identification of metabolites [16]. The TS and VS samples were analyzed as separate cohorts at two different time points (11/2008 and 1/2009). Data analysis was carried out using the statistical computing and graphics environment R [33]. For data normalization, each sample was divided by the sum over the measurements of the metabolites with known structure. Prior to analysis, data were transformed by log-2 scaling.

### Principal components analysis

Using unsupervised multivariate data analysis methods, we investigated whether the malignancy of tissues was among the main contributors to the variance of metabolites. To this end, the measurements of each identified metabolite were zero centered and principal components analysis (PCA) was performed. The rotation matrix that connects the 162 metabolites with the principal components was determined from the TS data. By multiplication with the rotation matrix, the principal components were calculated for the TS and VS. Finally, the principal components were investigated for changes between cancer and normal tissue samples. The significance of changes was assessed using Welch’s t-test.

### Detection of metabolite changes

We performed a supervised analysis and investigated each metabolite for change between cancer and normal tissues. A metabolic change was termed as detected, if there was a significant change in the TS (p < 0.05, Welch’s t-test, two-sided). A metabolic change was termed as validated, if there was a significant change in the VS in the same direction (p < 0.05, Welch’s t-test, one-sided). Using PROFILE clustering, the detected metabolite changes were analyzed in the context of the underlying metabolic pathways. PROFILE clustering is a bioinformatic method that groups metabolites with respect to the topologic distance within the metabolic network [14]. The distances for clustering are calculated from the network of enzymatic reactions as it is available from the KEGG database [34].

### Network reconstruction and visualization

A metabolic network was generated using Cytoscape [35] and MetScape, version 1.01 [36]. MetScape is a plug-in for Cytoscape that integrates reaction and pathway information from KEGG. The metabolites detected by GC-TOFMS were imported as the root for the network. MetScape automatically expanded the network with a depth of 2 reactions around each of the metabolites. Metabolites are mapped to nodes and reactions are mapped to edges connecting the nodes. The resulting network was manually curated by erasing nodes at the periphery of the network and by removing reactions that are not part of the human metabolism. Additional metabolites were added where the expansion depth was not sufficient. Information on the metabolic changes between breast cancer and normal tissues was imported as node attributes. The direction of regulation was visualized by the color of nodes and the fold change by the size of nodes.

### Marker metabolites and cancer detection

Separation of tumors (T) from normal breast tissues (NB) and tumors from adipose-rich tissues (NA) were investigated using receiver operator characteristics (ROC) curves. Metabolites that exceeded 80% sensitivity and specificity for both comparisons were considered as classifying. Classifying metabolites were divided in tumor markers (increased in T) and normal tissues markers (increased in NB and NA). Classifiers were constructed by the ratio of each possible combination of a tumor and a normal tissue marker. The quality of each classifier was investigated in an ROC analysis. For each classifier, an optimal cut-off point was determined by maximizing the sum of sensitivity and specificity. A voting system was applied counting each of the classifiers above the cut-off +1, and each of the classifier below the cut-off -1. Finally, each of the tissues was predicted to belong to the class with the majority of votes.

### Multiple random validation strategy

We investigated the performance of classifiers including 2, 3, …, 162 of the metabolites with known structure for the separation of cancer from normal tissues. For each of the metabolite numbers 200 random splits of the GC-TOFMS data were drawn. All training data sets included the same number of tumor and normal tissues (65 of each tissue type). Classification was performed by the nearest centroid method with respect to euclidean distance after feature selection based on Welch’s t-test. All analyses were conducted using the R package *cancerclass* that is available from the open source project *Bioconductor*[37]. 

## Competing interests

The authors have no competing interests to declare.

## Authors’ contributions

JB, CD and OF designed the study; RMS, JLG and MO contributed to the design of the study. CD, MD, CRE and UM collected and annotated tissue samples. CD, BMM, SFB and UM did the histopathological evaluation of the samples. GW and OF converted the GC-TOFMS spectra to metabolite data. JB analyzed the metabolite data; FK, BG and MH contributed to data analysis. JB wrote the manuscript. All authors read and approved the final manuscript.

## References

[B1] Hanahan D Weinberg RA Hallmarks of cancer: the next generation Cell 2011 144 646 674 10.1016/j.cell.2011.02.013 21376230

[B2] Warburg O On the origin of cancer cells Science (80- ) 1956 123 309 314 10.1126/science.123.3191.309 13298683

[B3] Tennant DA Durán RV Boulahbel H Gottlieb E Metabolic transformation in cancer Carcinogenesis 2009 30 1269 1280 10.1093/carcin/bgp070 19321800

[B4] Vander Heiden MG Cantley LC Thompson CB Understanding the Warburg effect: the metabolic requirements of cell proliferation Science (80- ) 2009 324 1029 1033 10.1126/science.1160809 PMC2849637 19460998

[B5] Rahman L Voeller D Rahman M Lipkowitz S Allegra C Barrett JC Kaye FJ Zajac-Kaye M Thymidylate synthase as an oncogene: a novel role for an essential DNA synthesis enzyme Cancer Cell 2004 5 341 351 10.1016/S1535-6108(04)00080-7 15093541

[B6] Griffin JL Shockcor JP Metabolic profiles of cancer cells Nat Rev Cancer 2004 4 551 561 10.1038/nrc1390 15229480

[B7] Kuhajda FP Fatty acid synthase and cancer: new application of an old pathway Cancer Res 2006 66 5977 5980 10.1158/0008-5472.CAN-05-4673 16778164

[B8] Glunde K Jie C Bhujwalla ZM Molecular causes of the aberrant choline phospholipid metabolism in breast cancer Cancer Res 2004 64 4270 4276 10.1158/0008-5472.CAN-03-3829 15205341

[B9] Menendez JA Lupu R Fatty acid synthase and the lipogenic phenotype in cancer pathogenesis Nat Rev Cancer 2007 7 763 777 10.1038/nrc2222 17882277

[B10] Kroemer G Pouyssegur J Tumor cell metabolism: cancer’s Achilles’ heel Cancer Cell 2008 13 472 482 10.1016/j.ccr.2008.05.005 18538731

[B11] Dang CV Rethinking the Warburg effect with Myc micromanaging glutamine metabolism Cancer Res 2010 70 859 862 10.1158/0008-5472.CAN-09-3556 20086171 PMC2818441

[B12] Tennant DA Durán RV Gottlieb E Targeting metabolic transformation for cancer therapy Nat Rev Cancer 2010 10 267 277 10.1038/nrc2817 20300106

[B13] Hilvo M Denkert C Lehtinen L Müller B Brockmöller S Seppänen-Laakso T Budczies J Bucher E Yetukuri L Castillo S Berg E Nygren H Sysi-Aho M Griffin JL Fiehn O Loibl S Richter-Ehrenstein C Radke C Hyötyläinen T Kallioniemi O Iljin K Oresic M Novel theranostic opportunities offered by characterization of altered membrane lipid metabolism in breast cancer progression Cancer Res 2011 71 3236 3245 10.1158/0008-5472.CAN-10-3894 21415164

[B14] Denkert C Budczies J Weichert W Wohlgemuth G Scholz M Kind T Niesporek S Noske A Buckendahl A Dietel M Fiehn O Metabolite profiling of human colon carcinoma–deregulation of TCA cycle and amino acid turnover Mol Cancer 2008 7 72 10.1186/1476-4598-7-72 18799019 PMC2569965

[B15] Denkert C Budczies J Kind T Weichert W Tablack P Sehouli J Niesporek S Könsgen D Dietel M Fiehn O Mass spectrometry-based metabolic profiling reveals different metabolite patterns in invasive ovarian carcinomas and ovarian borderline tumors Cancer Res 2006 66 10795 10804 10.1158/0008-5472.CAN-06-0755 17108116

[B16] Kind T Wohlgemuth G Lee DY Lu Y Palazoglu M Shahbaz S Fiehn O FiehnLib: mass spectral and retention index libraries for metabolomics based on quadrupole and time-of-flight gas chromatography/mass spectrometry Anal Chem 2009 81 10038 10048 10.1021/ac9019522 19928838 PMC2805091

[B17] Veronesi U Boyle P Goldhirsch A Orecchia R Viale G Breast cancer Lancet 2005 365 1727 1741 10.1016/S0140-6736(05)66546-4 15894099

[B18] Effects of chemotherapy and hormonal therapy for early breast cancer on recurrence and 15-year survival: an overview of the randomised trials Lancet 2005 365 1687 1717 15894097 10.1016/S0140-6736(05)66544-0

[B19] Chacón RD Costanzo MV Triple-negative breast cancer Breast Cancer Res 2010 12 Suppl 2 S3 10.1186/bcr2574 PMC2972557 21050424

[B20] Alvarez RH Present and future evolution of advanced breast cancer therapy Breast Cancer Res 2010 12 Suppl 2 S1 10.1186/bcr2572 21050422 PMC2972555

[B21] Michiels S Koscielny S Hill C Prediction of cancer outcome with microarrays: a multiple random validation strategy Lancet 2005 365 488 492 10.1016/S0140-6736(05)17866-0 15705458

[B22] Gatenby RA Gillies RJ Why do cancers have high aerobic glycolysis? Nat Rev Cancer 2004 4 891 899 10.1038/nrc1478 15516961

[B23] DeBerardinis RJ Mancuso A Daikhin E Nissim I Yudkoff M Wehrli S Thompson CB Beyond aerobic glycolysis: transformed cells can engage in glutamine metabolism that exceeds the requirement for protein and nucleotide synthesis Proc Natl Acad Sci U S A 2007 104 19345 19350 10.1073/pnas.0709747104 18032601 PMC2148292

[B24] Ahluwalia GS Grem JL Hao Z Cooney DA Metabolism and action of amino acid analog anti-cancer agents Pharmacol Ther 1990 46 243 271 10.1016/0163-7258(90)90094-I 2108451

[B25] Wang J Erickson JW Fuji R Ramachandran S Gao P Dinavahi R Wilson KF Ambrosio ALB Dias SMG Dang CV Cerione RA Targeting mitochondrial glutaminase activity inhibits oncogenic transformation Cancer Cell 2010 18 207 219 10.1016/j.ccr.2010.08.009 20832749 PMC3078749

[B26] Al-Kuraya K Schraml P Torhorst J Tapia C Zaharieva B Novotny H Spichtin H Maurer R Mirlacher M Köchli O Zuber M Dieterich H Mross F Wilber K Simon R Sauter G Prognostic relevance of gene amplifications and coamplifications in breast cancer Cancer Res 2004 64 8534 8540 10.1158/0008-5472.CAN-04-1945 15574759

[B27] Wu G Vance DE Choline kinase and its function Biochem Cell Biol 2010 88 559 564 10.1139/O09-160 20651826

[B28] Malcolm A Wright S Syms RRA Dash N Schwab M Finlay A Miniature mass spectrometer systems based on a microengineered quadrupole filter Anal Chem 2010 82 1751 1758 10.1021/ac902349k 20108919

[B29] Ouyang Z Cooks RG Miniature mass spectrometers Annu Rev Anal Chem (Palo Alto Calif) 2009 2 187 214 10.1146/annurev-anchem-060908-155229 20636059

[B30] Contreras JA Murray JA Tolley SE Oliphant JL Tolley HD Lammert SA Lee ED Later DW Lee ML Hand-portable gas chromatograph-toroidal ion trap mass spectrometer (GC-TMS) for detection of hazardous compounds J Am Soc Mass Spectrom 2008 19 1425 1434 10.1016/j.jasms.2008.06.022 18672381

[B31] Advanced interfaced micro-systems research for analysis of real-world clinical, food, environmental and waste samples (ARROWS) European FP7 Collaborative Project, http://www.arrows-online.eu

[B32] Sansone S Fan T Goodacre R Griffin JL Hardy NW Kaddurah-Daouk R Kristal BS Lindon J Mendes P Morrison N Nikolau B Robertson D Sumner LW Taylor C van der Werf M van Ommen B Fiehn O The metabolomics standards initiative Nat Biotechnol 2007 25 846 848 17687353 10.1038/nbt0807-846b

[B33] R: a language and environment for statistical computing, http://www.r-project.org

[B34] Kanehisa M Goto S Sato Y Furumichi M Tanabe M KEGG for integration and interpretation of large-scale molecular data sets Nucleic Acids Res 2012 40 D109 D114 10.1093/nar/gkr988 22080510 PMC3245020

[B35] Shannon P Markiel A Ozier O Baliga NS Wang JT Ramage D Amin N Schwikowski B Ideker T Cytoscape: a software environment for integrated models of biomolecular interaction networks Genome Res 2003 13 2498 2504 10.1101/gr.1239303 14597658 PMC403769

[B36] Gao J Tarcea VG Karnovsky A Mirel BR Weymouth TE Beecher CW Cavalcoli JD Athey BD Omenn GS Burant CF Jagadish HV Metscape: a Cytoscape plug-in for visualizing and interpreting metabolomic data in the context of human metabolic networks Bioinformatics 2010 26 971 973 10.1093/bioinformatics/btq048 20139469 PMC2844990

[B37] Budczies J Kosztyla D Cancerclass: an R package for development and validation of diagnostic tests from high-dimensional molecular data, Available from the Bioconductor repository. http://www.bioconductor.org

